# Diverse sediment microbiota shape methane emission temperature sensitivity in Arctic lakes

**DOI:** 10.1038/s41467-021-25983-9

**Published:** 2021-10-05

**Authors:** Joanne B. Emerson, Ruth K. Varner, Martin Wik, Donovan H. Parks, Rebecca B. Neumann, Joel E. Johnson, Caitlin M. Singleton, Ben J. Woodcroft, Rodney Tollerson, Akosua Owusu-Dommey, Morgan Binder, Nancy L. Freitas, Patrick M. Crill, Scott R. Saleska, Gene W. Tyson, Virginia I. Rich

**Affiliations:** 1 Department of Microbiology, The Ohio State University, 496W 12th Ave, Columbus, OH 43210 USA; 2 Department of Earth Sciences, University of New Hampshire, 56 College Road, Durham, NH 03824 USA; 3 Earth Systems Research Center, Institute for the Study of Earth, Oceans and Space, University of New Hampshire, 8 College Road, Durham, NH 03824 USA; 4 Department of Geological Sciences, Stockholm University, Stockholm, 106 91 Sweden; 5 Australian Centre for Ecogenomics, School of Chemistry and Molecular Biosciences, University of Queensland, Brisbane, 4072 Australia; 6 Civil & Environmental Engineering, University of Washington, 201 More Hall, Box 352700, Seattle, WA 98195-2700 USA; 7 Department of Environmental Science, University of Arizona, Tucson, AZ 85721 USA; 8 Department of Ecology and Evolutionary Biology, University of Arizona, Tucson, AZ 85721 USA; 9 Centre for Microbiome Research, Queensland University of Technology, 37 Kent St, Woolloongabba, QLD 4102 Australia; 10 Present Address: Department of Plant Pathology, University of California, Davis, One Shields Ave, Davis, CA 95616 USA; 11 Present Address: Center for Microbial Communities, Department of Chemistry and Bioscience, Aalborg University, Aalborg, 9220 Denmark; 12 Present Address: Department of Geological and Planetary Sciences, California Institute of Technology, Pasadena, CA 91106 USA; 13 Present Address: Parkland Hospital, 5200 Harry Hines Blvd., Dallas, TX 75235 USA; 14 Present Address: John C. Lincoln Health Network, 34975N North Valley Pkwy Ste 100, Phoenix, AZ 85086 USA; 15 Present Address: Energy and Resources Group, University of California, Berkeley, USA

**Keywords:** Metagenomics, Microbial ecology, Carbon cycle

## Abstract

Northern post-glacial lakes are significant, increasing sources of atmospheric carbon through ebullition (bubbling) of microbially-produced methane (CH_4_) from sediments. Ebullitive CH_4_ flux correlates strongly with temperature, reflecting that solar radiation drives emissions. However, here we show that the slope of the temperature-CH_4_ flux relationship differs spatially across two post-glacial lakes in Sweden. We compared these CH_4_ emission patterns with sediment microbial (metagenomic and amplicon), isotopic, and geochemical data. The temperature-associated increase in CH_4_ emissions was greater in lake middles—where methanogens were more abundant—than edges, and sediment communities were distinct between edges and middles. Microbial abundances, including those of CH_4_-cycling microorganisms and syntrophs, were predictive of porewater CH_4_ concentrations. Results suggest that deeper lake regions, which currently emit less CH_4_ than shallower edges, could add substantially to CH_4_ emissions in a warmer Arctic and that CH_4_ emission predictions may be improved by accounting for spatial variations in sediment microbiota.

## Abstract (web summary)

Arctic lakes are strong and increasing sources of atmospheric methane, but extreme conditions and limited observations hinder robust understanding. Here the authors show that microbes in the middle of Arctic lakes have elevated methane producing potential, and are poised to release even more in the future.

## Introduction

At high latitudes, lakes and ponds are recognized as a large and understudied source of methane (CH_4_)^1–4^, a radiatively important trace gas. Post-glacial lakes (formed by glaciers and receding ice sheets, leaving mineral-rich sediments) represent the largest lake area at high latitudes^5^. Because of their areal extent, these lakes contribute to approximately two-thirds of the model-predicted natural CH_4_ emissions above 50°N latitude^1^. Their geochemistry and emissions are distinct from thermokarst lakes formed by permafrost thaw^6^. With warming, permafrost thaw, and predicted increased precipitation, northern lakes are expected to receive more terrestrially derived carbon, likely increasing their carbon dioxide (CO_2_) and CH_4_ emissions^7,8^.

Ebullition commonly accounts for >50%, sometimes >90%, of the CH_4_ flux from post-glacial lakes, with the remainder primarily attributed to diffusion-limited hydrodynamic flux^9,10^. Ebullition moves CH_4_ rapidly from sediments directly to the atmosphere, typically bypassing microbial CH_4_ oxidation in the water column^11^. Incoming short-wave radiation and sediment temperature have been identified as strong predictors of ebullitive CH_4_ emission from sub-arctic post-glacial lakes on an annual basis, with higher temperatures increasing emissions during the ice-free season^2,12^. However, the extent and drivers of spatial variability in this temperature response, particularly within lakes, are poorly understood.

To address this knowledge gap, we analyzed ebullitive CH_4_ emissions over a 6-year period and collected underlying sediment cores in July 2012 from the littoral (edge) and pelagic (middle) locations of two shallow post-glacial lakes, Mellersta Harrsjön and Inre Harrsjön (Supplementary Fig. 1, Supplementary Table 1). These lakes are part of the Stordalen Mire complex, a hydrologically interconnected, discontinuous permafrost ecosystem encompassing post-glacial lakes and a mosaic palsa/wetland in approximately equal portions^13^. The lakes contribute ~55% of the total Stordalen Mire ecosystem CH_4_ loss^2^, and are model sites for studying ebullitive CH_4_ emissions via inverted funnel bubble traps at the lake surface^9,12,14^. These ebullitive flux measurements were collected for the six summers from 2009 to 2014^12,14^ every 1–3 days^9^. Here, we analyzed 5126 ebullitive CH_4_ emission measurements (Supplementary Table 2) from this previously published dataset for spatial patterns (edge vs. middle), and we linked these patterns to analyses of the microbiota and biogeochemistry in the underlying sediments.

## Results and discussion

### Spatial variation in ebullitive CH_4_ temperature sensitivity

**Fig. 1:**
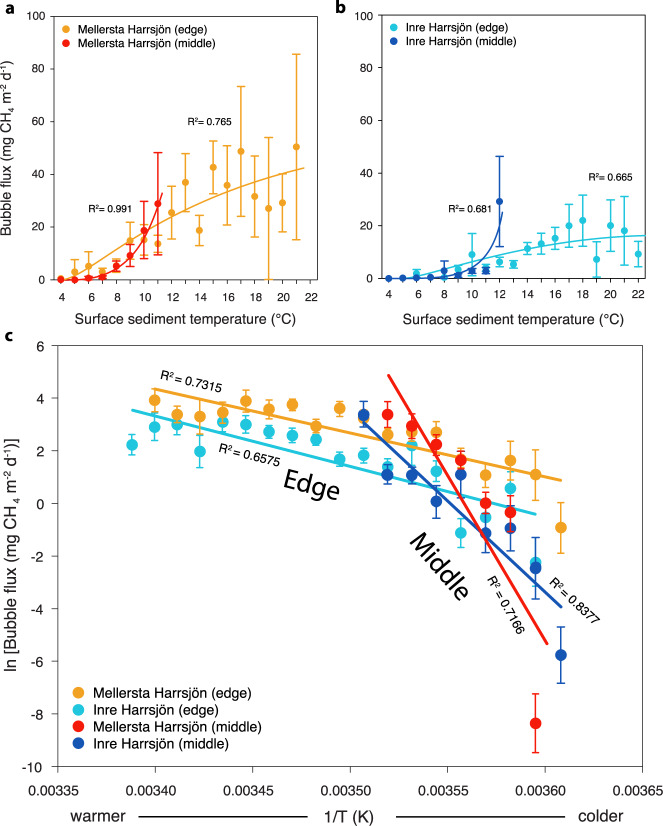
Temperature responsiveness of ebullitive methane flux from two post-glacial lakes. Ebullitive CH_4_ flux as a function of surface sediment temperature (data were binned in 1 °C intervals; see Methods) from June to September 2009–2014 for the edge vs. middle regions of **a** Lake Mellersta Harrsjön (MH) (MH edge—*n* = 1581, MH middle—*n* = 795 independent ebullitive CH_4_ flux measurements) and **b** Lake Inre Harrsjön (IH) (IH edge—*n* = 2318, IH middle—*n* = 432 independent ebullitive CH_4_ flux measurements). Error bars in **a** and **b** are 95% confidence intervals, fit lines are 2nd-degree polynomials, and points are means. **c** Arrhenius plots of the data in **a** and **b** (*n* = 5126 independent ebullitive CH_4_ flux measurements); ln(bubble CH_4_ flux) vs. the inverse surface sediment temperature in K. Points are means and error bars are 95% confidence intervals. Data are color-coded by the lake and by edge (littoral) and middle (pelagic) zones.

Previous work has shown that annual ebullitive emissions are consistently higher from these lakes’ shallow littoral zones than their deeper pelagic zones^9,15^, as expected since the shallow sediments experience higher temperatures for longer periods and also receive more substrate input from aquatic vegetation^16^. However, assessing the temperature sensitivity of ebullition for the two lake zones in this study revealed a previously unnoticed significant difference, with ~3–5-fold higher temperature sensitivity in lake middles relative to edges (Fig. 1, Supplementary Table 3). The statistical significance of these differences was consistent across all edge-to-middle comparisons (within and between lakes) and was generally also robust to re-analyses considering three subsets of the data (removal of edge data at temperatures above those experienced by middles and removal of potential outliers at the highest and lowest temperatures experienced by lake middles, respectively), with one exception: Inre Harrsjön edges and middles were not statistically significantly different when higher temperature edge data were removed (Supplementary Table 3, Supplementary Fig. 2). Predicted future emissions from post-glacial subarctic lakes are based on current measurements of temperature responsiveness^1^, which are dominated by ebullitive flux data from shallow lake zones because those locations currently experience a longer period of sufficient warmth for seasonal emissions than lake middles (~3 months relative to ~1 month)^2^. If, as suggested here by our spatially resolved emissions data, temperature responsiveness is substantively higher in the deeper sediments, then, as deeper regions warm and remain heated for longer before cooling off in the fall, future lake emissions would be greater than currently predicted. Thus, accurate CH_4_ emission predictions rely on understanding the spatial heterogeneity and underlying causes of this temperature responsiveness.

Ebullition is controlled by CH_4_ production (which is in turn driven by redox, substrates, temperature, and microbiota), consumption (driven by redox and microbiota)^17–19^, and the physics of bubble formation and escape (determined by sediment texture and overlying hydrostatic pressure, which is largely controlled by atmospheric conditions)^2,15^. Therefore, the edge-to-middle difference in temperature responsiveness of CH_4_ ebullition could be partly due to differences in physicochemical characteristics (e.g., sediment texture, pressure, and redox), substrates (e.g., organic carbon), and/or microbiota (abundance, composition, and/or activity)^20^. Although differences in sediment texture were observed between the lake edge and middle in Mellersta Harrsjön, these differences were not consistent between lakes (Supplementary Fig. 3, Supplementary Table 4). Our previous work has shown higher and more variable ebullition rates during periods of dropping atmospheric pressure, but there were no differences in edge vs. middle locations^9^. In terms of redox, we expect concentrations of terminal electron acceptors to be low, as the likely source would be runoff^21^, and total sulfur and nitrogen did not correlate with ebullition rates by lake or location^15^. In terms of measured substrates, carbon:nitrogen (C:N) ratios and bulk δ^13^C_TOC_ (indicative of vegetation composition) did not vary from edges to middles. Total organic carbon (TOC) varied by the lake, with similar concentrations observed between lake edge and middle in Mellersta and appreciably higher TOC in middle sediments in Inre Harrsjön. Carbon quality, as assessed by visual comparisons of organic matter composition, revealed coarse, less decomposed detritus gyttja (organic-rich, peat-derived mud) in the edge sediments of both lakes, while middle sediments were characterized by fine-grained, generally more decomposed detritus gyttja^15^. Thus, higher temperature responsiveness occurred where there was lower potential substrate quality, suggesting that substrate differences do not readily explain patterns in CH_4_ emission responses to temperature in edge vs. middle lake locations, although more detailed substrate analyses could further evaluate this in the future.

### Spatial differences in sediment microbial communities

**Fig. 2:**
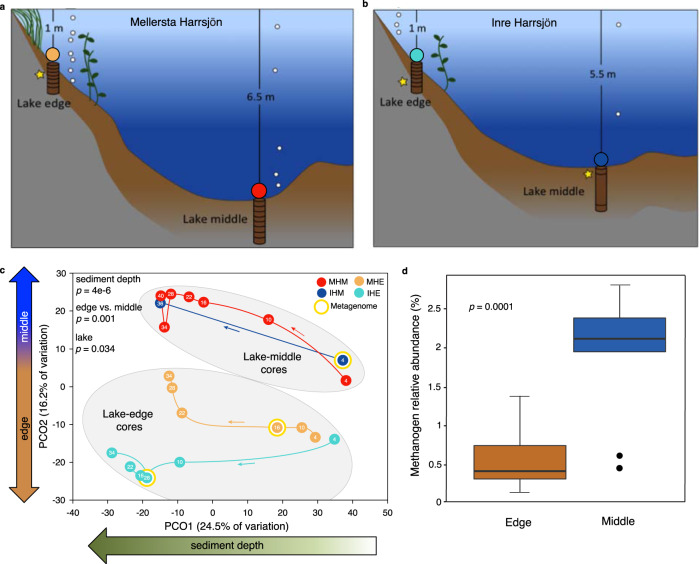
Lake sediment bacteria and archaea in two post-glacial lakes. **a**, **b** Schematic overview of lakes and cores collected for DNA sequencing analyses, with core subsections indicated by horizontal lines. Cores in each lake are referred to as Lake edge or Lake middle, with overlying water depth as indicated, and the four colored circles are used to distinguish each core and/or lake location throughout the figures. Yellow stars indicate cores and depths targeted for shotgun metagenomics. **c** Principal coordinates analysis (PCoA) of microbial community composition across samples (each core subsection, *n* = 21), based on 16S rRNA gene amplicon abundances of microbial operational taxonomic units (OTUs); circles represent samples, and samples in closer proximity have more similar microbial community composition. Thin arrows along colored lines indicate increasing depth within each core. *p*-Values from one-way PERMANOVA indicate how significantly microbial community composition differed according to the indicated categorical variable (significant if *p* < 0.05). **d** Percent relative abundance of OTUs identified as methanogens in 16S rRNA gene amplicon data in lake edges compared to lake middles (*p*-value from two-tailed Student’s *t*-test, significant if *p* < 0.05), *n* = 21 biologically independent samples. Lines in boxes depict the median, boxes indicate 75th percentile, whiskers 95th percentile, and points are outliers.

Next, we sought to characterize differences in the microbiota that could contribute to the observed temperature response patterns in CH_4_ emissions. We first used a 16S rRNA gene amplicon sequencing approach to characterize microbial community composition from the edge and middle cores from each lake (Fig. 2A, B, Supplementary Table 5). Although microbial community composition differed most significantly by depth within the sediment (Supplementary Fig. 4, Supplementary Table 6), as is typical for aquatic sediments^22^, significant differences between lake edges and middles (Fig. 2C, PERMANOVA *p* = 0.001) revealed shared spatial patterns in microbiota and ebullitive CH_4_ flux measurements, suggesting that microbiota could contribute to the observed temperature sensitivity in CH_4_ emissions.

While total microbial abundances correlated most strongly with depth in the sediment and did not exhibit edge vs. middle differences (*p* = 0.15, Supplementary Fig. 5, Supplementary Table 7), an exploration of functional guilds revealed multiple significant edges vs. middle differences. Methanogens (defined here as populations from known methanogenic clades^23^, Supplementary Table 5) were significantly more relatively abundant in lake middles than edges (Fig. 2D, ANOVA *p* = 0.0001). Combining relative abundances and quantitative polymerase chain reaction (qPCR) absolute abundances as a proxy for methanogen total abundances showed no significant difference between lake edges and middles (*p* = 0.94), likely due to the strong sediment-depth patterns of total cell numbers (qPCR abundances) in combination with the low relative abundances of methanogens (0.3–2.3%, consistent with other studies^24–27^). Syntrophs have been shown to be upstream regulators of methane production^28–31^, and they can be obligately mutualistic with methanogens, for example by producing hydrogen used in hydrogenotrophic methanogenesis. While syntrophy does not exhibit a strong phylogenetic signal^32^, precluding its robust quantification here as a functional guild, the Syntrophaceae lineage, whose syntrophic potential was supported by reconstruction of a metagenome-assembled genome (MAG) (see below), did show significantly higher relative abundances in lake middle sediments relative to edges (*p* = 0.047). Aerobic methanotrophs, which are posited to have minimal impact on ebullitive CH_4_ loss due to rapid bubble movement through sediment^11^, were confined to the surface sediment layers as expected (Supplementary Table 8) and did not differ significantly in composition or relative abundance between edges and middles (ANOVA *p* = 0.76). Anaerobic methanotroph (ANME archaeal) abundances differed significantly between lake edges and middles (ANOVA *p* = 0.014) and were approximately one order of magnitude higher in edge sediments (Supplementary Tables 8 and 9). Although this could suggest that increased anaerobic methane oxidation in the edge sediments could contribute to the observed differences in temperature sensitivity, these ANME archaea comprised only 0.1% of the community on average (range 0.0–0.6%, Supplementary Tables 5 and 8), and again, ebullition is expected to largely bypass methane oxidation. Still, future work to further constrain ANME activity at different temperatures^33,34^ may help to elucidate the significance of the observed patterns in ANME abundance.

### Sediment incubations and modeling of CH_4_ production

**Fig. 3:**
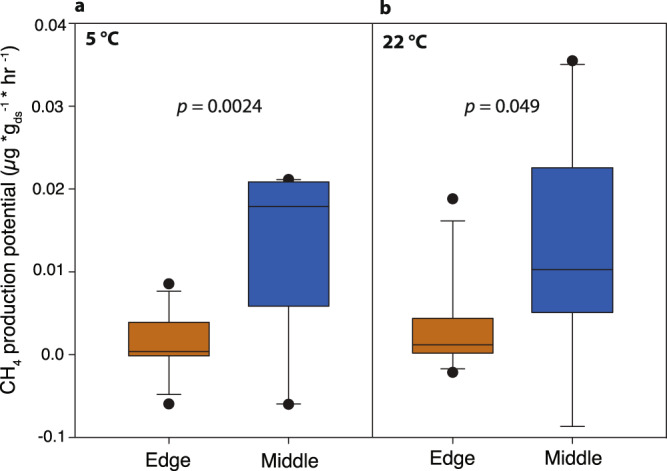
Methane production from anaerobic laboratory incubations of lake sediments. Sediments were collected from edges and middles of lakes Inre Harrsjön and Mellersta Harrsjön in 2012 and 2013 (see Methods) and incubated at **a** 5 °C (*n* = 12 independent incubation experiments) and **b** 22 °C (*n* = 12 independent incubation experiments). Headspace CH_4_ concentrations were measured daily for 5 days and average daily CH_4_ fluxes were calculated for each sample. Lines in boxes depict the median, boxes indicate 75th percentile, whiskers 95th percentile, and points are outliers. ds dry sediment. *p*-Values for both **a** and **b** are from one-way ANOVA edge vs. middle comparisons (significant if *p* < 0.05).

To more directly evaluate the CH_4_ production potential of the microbial communities in these sediments at different temperatures, we performed 48 ex situ anaerobic incubations of edge and middle sediments collected in 2012 (linked directly to our microbial and biogeochemical data) and 2013 (from the same four core locations) (Supplementary Table 10). These incubations at 5 and 22 °C showed that the lake-middle sediments had significantly higher CH_4_ production potentials than lake-edge sediments at both temperatures (Fig. 3), paralleling their higher methanogen and syntroph relative abundances and indicating that the lake-middle methanogens can remain metabolically active at higher temperatures, despite never yet experiencing them in situ. However, it is important to note that a difference in temperature responsiveness (i.e., different CH_4_ production potentials at 5 °C, relative to 22 °C) was not observed for the edge or middle sediments (ANOVA *p* = 0.38 for edges, *p* = 0.91 for middles), as might have been expected from the in situ ebullition data (Fig. 1). One potential explanation for this is that the ex situ incubations measured relatively immediate CH_4_ production when the entire sediment was maintained at a certain temperature, whereas the in situ measurements linked temperatures at the sediment/surface interface with ebullitive CH_4_ flux measurements at the lake surface. Thus, the measured in situ temperatures were somewhat disconnected from the temperatures (and times) of CH_4_ production, and their associated flux measurements represent CH_4_ production potential integrated over the time scales of bubble generation and flow through the sediment and water column, as observed over multiple years.

Since lake surface ebullition measurements represent CH_4_ production integrated across depths in the underlying sediment, we applied isotope and mass balance calculations to partition CH_4_ production to its likely source depths, in order to link CH_4_ production to depth-discrete microbiota. Based on stable carbon isotope values and porewater concentrations of CH_4_ and dissolved inorganic carbon (DIC), we inferred total CH_4_ loss (fugitive CH_4_) at each depth interval (Supplementary Table 4). This fugitive CH_4_ production was correlated with microbiota from the same depths, revealing a significant correlation between overall microbial community composition and fugitive CH_4_ (Mantel *p* = 0.016) and between fugitive CH_4_ and the relative abundances of specific microbial lineages, including Phycisphaerae, Thermoplasmata, and Aminicenantes (Supplementary Tables 6 and 11). All of these lineages have representative MAGs with metabolic reconstructions discussed further below.

### Microbial metabolic predictions and ties to biogeochemistry

To more specifically investigate links between CH_4_-associated microbial functional guilds and CH_4_ chemistry, we identified multiple known CH_4_-cycling clades in the 16S rRNA gene amplicon data and applied targeted metagenomic sequencing to a subset of samples to examine diagnostic genes for CH_4_ cycling (and to assemble genomes for metabolic pathway reconstructions, discussed further below). From the metagenomes, using hidden Markov models (see Methods), we recovered 5470 sequencing reads with high similarity to phylogenetically diverse functional genes indicative of CH_4_ production (*mcrA*) and consumption (*pmoA*) potential (Supplementary Fig. 6, Supplementary Table 12). The *mcrA* data, together with 16S rRNA gene abundances of specific lineages of known hydrogenotrophic and acetoclastic methanogens, support αC isotopic calculations in indicating that hydrogenotrophic methanogenesis was the dominant methanogenic pathway in these lake sediments (Supplementary Fig. 7).

**Fig. 4:**
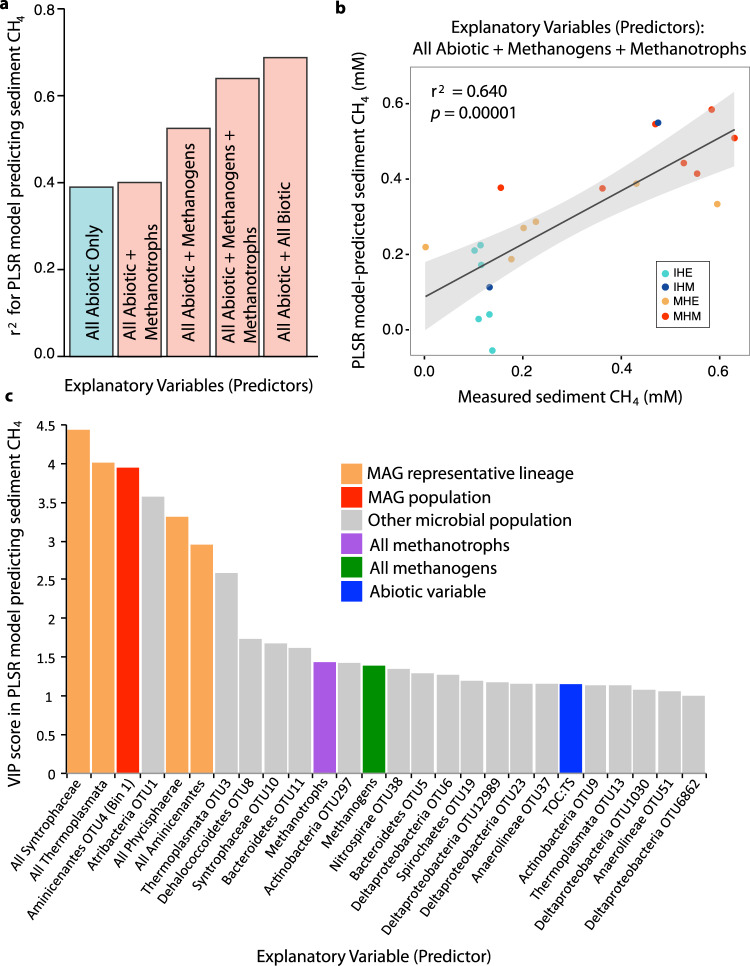
Partial least squares regression (PLSR) statistical modeling to predict sediment CH_4_ concentrations. PLSR analyses tested the ability of different suites of explanatory variables to predict measured sediment CH_4_ concentrations in the four cores from 2012 across depths (*n* = 21); in all models, all measured abiotic variables (except those related to CH_4_ concentrations, see Methods) were included as explanatory variables, and biotic variables were added as indicated. Biotic variables included relative abundances of specific OTUs and/or summed OTU abundances grouped by taxonomy or predicted metabolism (as indicated), from 16S rRNA gene amplicon data. **a** Correlation coefficient (*r*^2^) for PLSR models predicting sediment CH_4_ using different combinations of explanatory variables. Each bar represents a single underlying data point, with the value of that point indicated by the bar height along the *y*-axis. **b** Linear regression of measured and model-predicted sediment CH_4_, considering all abiotic variables and methanogen and methanotroph abundances as explanatory variables; error band represents 95% confidence interval; each point is a sample, colored by core. **c** For the model with the highest *r*^2^ (rightmost in panel **a**), all significant explanatory variables are shown (VIP scores > 1, *n* = 26 sig*n*ificant explanatory variables out of 153 total variables considered). VIP scores show the relative contribution of each variable to the model, with higher VIP scores indicating a more significant contribution. Each bar represents a single underlying data point, with the value of that point indicated by the bar height along the *y*-axis.

Using partial least squares regressions (PLSR) and multiple linear regression (MLR) analyses, we predicted porewater CH_4_ concentrations from methanogen and methanotroph relative abundances, as measured via 16S rRNA gene amplicon sequencing data. Although the modeled fugitive CH_4_ calculations might have provided a better depth-resolved link to CH_4_ production than the porewater CH_4_ concentrations analyzed here, we opted to use two direct measurements (microbiota and porewater CH_4_) in these statistical models, rather than essentially modeling a model. When using either PLSR or MLR to predict porewater CH_4_ concentrations, a better prediction was achieved when both depth-resolved abiotic variables (i.e., depth, TOC, DIC, ^13^C_TOC_, S, and TOC:TS, see Methods) and the relative abundances of predicted CH_4_-cycling organisms were included (PLSR: *r*^2^ = 0.640, *p* = 0.00001, MLR: adjusted *r*^2^ = 0.752, *p* = 0.0003), relative to including the abiotic variables alone (PLSR: *r*^2^ = 0.390, *p* = 0.002, MLR: adjusted *r*^2^ = 0.532, *p* = 0.0004) (Fig. 4A, B, Supplementary Table 13). These results suggest that direct measurements of microbial abundances could contribute to more accurate predictions of future CH_4_ emissions, consistent with previous statistical models that have linked specific microbiota to C- and/or CH_4_-cycling dynamics in marine ecosystems and thawing permafrost peatlands^35–39^.

By expanding our PLSR analyses to consider the full microbial community, in addition to known CH_4_ cycles, our ability to predict CH_4_ concentrations improved further. This analysis considered the following groupings of 16S rRNA gene abundances as explanatory variables for the prediction of porewater CH_4_ concentrations: (1) each operational taxonomic unit (OTU) at >1% relative abundance in any sample (Supplementary Table 5), (2) summed lineage abundances of all bacteria and archaea (mostly at the phylum or class levels, see Supplementary Fig. 4 for groupings), and (3) summed abundances of the most highly resolved lineage representative in the amplicon data for each MAG (a population genome computationally reconstructed from shotgun metagenomic community DNA sequencing data, Supplementary Table 14). In two cases (for Aminicenantes MAG Bin 1/OTU 4 and a Methanomassiliicoccales-related Thermoplasmata, MAG Bin 16/OTU 27), a MAG was linked directly to a specific OTU in the amplicon data through a co-binned 16S rRNA gene sequence in the MAG, such that the MAG relative abundance could be inferred from the amplicon data. In all other cases, the summed abundances of amplicon OTUs in the same lineage as the MAG were used as proxies for MAG abundances. A total of 153 potential explanatory variables were considered in this PLSR analysis, 26 of which, including methanogen and methanotroph abundances, were identified as significant predictors of porewater CH_4_ concentrations (Fig. 4C).

Four of the top five microbial groups most predictive of porewater CH_4_ concentrations in the PLSR analysis were lineages for which we were able to reconstruct a MAG (Fig. 4C, Supplementary Tables 15 and 16), thus organization into MAGs helped to unravel the specific metabolic processes most predictive of carbon chemistry. In total, five MAGs were reconstructed with >85% completeness and <6% contamination (Supplementary Discussion). The best overall predictor of porewater CH_4_ concentrations was the Syntrophaceae class of Deltaproteobacteria, which was considered in the PLSR analysis as the summed abundance of all OTUs in this clade. Consistent with a syntrophic metabolism, including hydrogen production (e.g., in support of hydrogenotrophic methanogenesis, which is the dominant methanogenic pathway in these sediments, as described above), the Syntrophaceae MAG revealed 15 hydrogenase-associated genes, along with the capacity to ferment diverse carbon compounds (particularly carbon-sulfur compounds), with the added potential capacity for respiration (see Supplementary Discussion).

Though the Syntrophaceae lineage was overall most predictive of porewater CH_4_ concentrations, the most significant predictive single OTU was a member of the candidate phylum Aminicenantes, which we also recovered as a MAG. While this lineage has been previously predicted to be fermentative, saccharolytic, and/or aerobic^40–42^, our lake sediment genome revealed the metabolic potential for several C1 metabolic processes, including methylotrophy through the assimilation of methylamines, methane-thiols, and/or dimethylsulfide, similar to previous recoveries of complete Wood–Ljungdahl pathways for C1 metabolism via carbonyl and methyl pathways in this lineage^43^. The predicted capacity for methylotrophy could explain the strong correlation between Aminicenantes relative abundance and porewater CH_4_ concentrations.

The relative abundances of two other lineages with MAGs, the Thermoplasmata (a group of Archaea) and Phycisphaerae (a class of Planctomycetes bacteria), were also strongly predictive of both porewater CH_4_ concentrations in the PLSR analysis and of calculated fugitive CH_4_ in linear regressions (Supplementary Tables 11 and 16). Phylogenetic analyses showed that the Thermoplasmata Bin 19 MAG was derived from a divergent member of the Thermoplasmatales order, and it encodes the capacity for CO_2_ production from a formate, along with peptide and amino acid degradation (as previously indicated^44^) and complex carbon degradation. Our recovered Phycisphaerae population genome appears to have the capacity to metabolize a wide variety of complex carbon compounds, potentially via fermentation, consistent with previous predictions for the Planctomycetes phylum^45^. While direct ties to CH_4_ are not obvious in these two genomes, we speculate that their contributions to overall carbon cycling, potentially including through fermentative contributions of CO_2_ as a substrate, may be driving these strong correlations with both CH_4_ concentrations and modeled fugitive CH_4_.

Interestingly, the only lineage represented by a MAG that was not a significant predictor of porewater CH_4_ concentrations in the PLSR analysis was classified by its 16S rRNA gene sequence as a member of the Methanomassiliicoccales, an archaeal lineage presumed to consist exclusively of obligate H_2_-dependent methylotrophic methanogens^46,47^. We hypothesized that this lake sediment population did not have the capacity for methanogenesis, as we did not recover genes from the methanogenesis pathway in this 95% complete genome. Further analysis of this MAG, reported in a separate paper^48^, revealed that this population is not a member of the Methanomassiliicoccales but rather is part of a new order basal to the Methanomassiliicoccales within the Thermoplasmata. All 12 MAGs that we analyzed from this new order and related lineages (including 11 MAGs from other ecosystems, mostly anoxic sediments) were found to lack >20 methanogenesis biomarker genes that are present in Methanomassiliicoccales genomes, including *mcrA*. Instead, the lake sediment MAG reported here is predicted to conserve energy through amino acid metabolism^48^. While *mcrA* sequences that were putatively phylogenetically assigned to Methanomassiliicoccaceae were recovered from metagenomic reads in our GraftM analysis here (Supplementary Table 12), these genes were presumably derived from other OTUs classified as Methanomassiliicoccaceae in our amplicon data, some of which may be true methanogens. These results highlight the value of reconstructing near-complete genomes for a better understanding of metabolism, along with the limitations of using 16S rRNA gene sequences to infer metabolic processes.

In conclusion, we found significant differences in the slope of the temperature vs. CH_4_ flux relationship between sub-arctic lake edges and middles, suggesting that radiative forcing (temperature) and a concomitant increase in microbial metabolic rates are not the only primary controls on CH_4_ emissions. These edge vs. middle differences were shared by underlying sediment microbial communities, suggesting that differences in sediment microbial community composition (i.e., not simply differences in microbial activity) contribute to spatial differences in the response of CH_4_ emissions to increasing temperature. Specifically, we observed significant differences in microbial community composition between lake edges and middles, including significantly higher methanogen and Syntrophaceae abundances in lake middles relative to edges, and CH_4_ emissions from lake middle sediments were significantly higher than from lake edges when incubated at the same temperatures. In addition, the relative abundances of CH_4_-cycling organisms and their reconstructed population genomes (MAGs) were significantly better predictors of sediment CH_4_ concentrations than abiotic variables alone. Syntrophic lineages, which can generate the hydrogen required for hydrogenotrophic methanogenesis, and lineages capable of C degradation to CO_2_ (also potentially “upstream” of methanogenesis) were also predictive of sediment CH_4_ concentrations. Together, these results suggest that when lake middles reach the temperatures of lake edges, they may emit even more CH_4_ than the lake edges currently do, such that our projected future CH_4_ emissions may be underestimating contributions from subarctic lakes, and that knowledge of microbial community composition and metabolism could improve these predictions. Future investigations that consider the combined effects of microbiota, carbon quality, and temperature on lake CH_4_ emissions, including further exploration of sediment depth-resolved contributions to total CH_4_ emissions across multiple locations and years, will help to provide a more comprehensive understanding of spatiotemporal controls on global CH_4_ emissions.

## Methods

### Field site and sample collection

Stordalen Mire is a subarctic peatland complex located 10 km east of Abisko in northern Sweden (68°21′N, 19°02′E). Lakes Mellersta Harrsjön and Inre Harrsjön are 1.1 and 2.3 ha in area, reaching maximum depths of 7 and 5 m, respectively^49^. These lakes are post-glacially formed, and, unlike some thermokarst lakes at high latitude, they do not have underlying permafrost or seeps that emit geologic CH_4_; the CH_4_ is biologically derived^9,21^. Mellersta Harrsjön receives water from a small stream, while Inre Harrsjön is fed through groundwater and runoff from the surrounding mire. Ebullitive and diffusion-limited CH_4_ emissions from these lakes have been documented using floating funnels and chambers distributed across the lakes, and sampled frequently^2,9,12^. Ebullition varies spatially with higher emissions from shallow zones and in the presence of plants^9,15^.

We collected quadruplicate sediment cores (four cores from two locations in each of two lakes: Mellersta Harrsjön edge (68°357832′N, 19°042046′E) and middle (68°358291′N, 19°042132′E) and Inre Harrsjön edge (68°357880′N, 19°048525′E) and middle (68°358418′N, 19°045650′E)) on July 10 and 18, 2012 at the Stordalen Mire nature reserve, a research site near Abisko, northern Sweden (Supplementary Table 1). Samples were taken from cores (as described below) along a depth gradient (ranging from 4 to 40 cm) for geochemical measurements and microbial DNA sequencing data.

### Geochemical data collection and analysis

For each set of four cores, we sampled the first core for sediment C, N, and S (weight percent), percent TOC, and bulk sediment ^13^C_TOC_ and ^15^N_TOC_. Samples of 1 cm^3^ were taken in 6 cm increments from the top of the core to the bottom. The samples were then dried, ground, and split into an untreated sample for total carbon (C) and an acidified TOC sample. Details regarding sample preparation for measurement on a Perkin Elmer 2400 Series II CHNS/O Elemental Analyzer at the University of New Hampshire (UNH) were described previously^15^. Repeatability error was established by analyzing replicate samples and calculating the standard deviation. Duplicate samples were run approximately every 10 samples. Potential outliers were also run in duplicate. Isotopic analysis was performed by combusting dried sediment samples in a Costech ECS 4010 elemental analyzer coupled to a Thermo Trace GC Ultra isotope ratio mass spectrometer (IRMS), based on calibration with acetanilide, Atlantic cod, black spruce needles, sorghum flour, corn gluten, NIST 1515 apple leaves, and tuna muscle standards (UNH Stable Isotope Lab). In 2013 we also collected sediment cores in the same locations in these lakes. We report sediment textural analyses from these cores as % sand, % silt, and % clay (Supplementary Table 4). Those samples were dried and run through a laser particle size analyzer (Malvern Mastersizer 2000).

The second replicate core was used for quantifying total CH_4_ in the core sediment reported in μM. After coring, we pulled 2 cm^3^ sediment plugs using cut plastic syringes through pre-drilled holes cut at 4 cm increments along the core liner. The sediment plugs were transferred to 30 ml serum vials containing 5 ml of 2 M NaOH, capped quickly, and shaken^50,51^. After sitting overnight then heating for 1 h at 60 °C, the headspace of the vials was analyzed for CH_4_ using a Shimadzu GC-2014 gas chromatograph (GC) with a flame ionizing detector^9^. The CH_4_ measured represents the total, that is, nearly all of the CH_4_ dissolved in the water from the sediment plug and any bubbles that may have been trapped in the sediment. The remaining sediment samples in the vials were weighed and dried to constant weight to determine the mass of water in the samples to be used for calculating the CH_4_ concentration in μM.

The third replicate core was used for the measurement of DIC. Rhizon samplers were inserted every 2 cm through pre-drilled holes in the core and a vacuum was pulled with a 30 ml polypropylene syringe. The first ~1 ml of sediment water was discarded because of contamination with DI water. After 10 ml of sediment pore water was collected, it was injected into a 30 ml evacuated serum vial with 1 ml 30% H_4_PO_4_ solution. This caused forms of inorganic C in the water to form CO_2_. A headspace sample was then extracted and run on an infrared gas analyzer to determine the CO_2_ concentration.

Porewater isotopic composition was determined in samples from cores collected in the same locations in 2014. Methods were described previously^35^. Briefly, sample vials that were collected for CH_4_ and DIC were acidified with 0.5 ml of 21% H_3_PO_4_ and brought to atmospheric pressure with helium. The sample headspace was analyzed for δ13C of CH_4_ and CO_2_ on a continuous-flow Hewlett-Packard 5890 gas chromatograph (Agilent Technologies) at 40 °C coupled to a FinniganMAT Delta S isotope ratio mass spectrometer via a Conflo IV interface system (Thermo Scientific).

Methods for measuring ebullition and water temperature have been described previously, and the ebullitive CH_4_ flux and temperature data analyzed here are a subset of those previously reported^9^. In brief, measurements of CH_4_ bubble flux during the ice-free season (June to September) have been ongoing at these lakes since 2009. A total of 40 bubble traps (here, 22 traps), distributed in a depth-stratified sampling scheme were sampled frequently (every 1–3 days). For this study, averages of CH_4_ bubble flux were calculated for each lake by binning data from the edge (shallower water, littoral) and middle (deeper water, pelagic) areas separately in 1 °C interval (total of 4–22 °C) of corresponding surface sediment temperature. For this, we used flux and temperature data collected from 2009-2014. Water and surface sediment temperatures were measured in profiles continuously, using intercalibrated Onset HOBO v22 loggers, as previously described^9^ (data are available here: https://bolin.su.se/data/). The binned flux data were used to construct Arrhenius equations in order to investigate differences in temperature response on the ebullition from the edge and middle areas.

### DNA extraction and 16S rRNA gene sequencing

A fourth replicate core was collected for DNA extraction. After coring, we pulled 2 cm^3^ sediment plugs using cut plastic syringes through pre-drilled holes cut at 4 cm increments along the core liner. Samples were immediately put in Eppendorf tubes and placed in a cooler until returned to the research station where they were stored at −80 °C until extraction.

For DNA extraction from each core depth range, 0.25 g of sediment was collected under sterile conditions and added to a MoBio PowerSoil DNA Isolation Kit (MoBio, Inc., Carlsbad, CA, USA). DNA was extracted according to the manufacturer’s instructions. PCR amplification and sequencing were performed at the Environmental Sample Preparation and Sequencing Facility at Argonne National Laboratory, in accordance with previously described protocols^52–54^. Briefly, 515F and barcoded 806R primers with Illumina flowcell adapter sequences were used to amplify the V4 region of bacterial and archaeal 16S rRNA genes^55^. Each 25 μl PCR reaction contained 12 μl of PCR water (MoBio, Inc., Carlsbad, CA, USA), 10 μl of 1 × 5 PRIME Hot Master Mix (5 PRIME Inc., Bethesda, MD, USA), 1 μl each of F and R primers (5 μM concentration, 200 pM final), and 1 μl of template DNA. PCR cycling conditions were as follows: 94 °C for 3 min, 35 cycles of [94 °C for 45 s, 50 °C for 60 s, and 72 °C for 90 s], 72 °C for 10 min. A PicoGreen assay (Life Technologies, Grand Island, NY, USA) was used to measure amplicon concentrations. Equimolar concentrations for each barcoded sample were combined and then cleaned with the UltraClean PCR Clean-Up Kit (MoBio Inc., Carlsbad, CA, USA) and then quantified using the Qubit (Invitrogen, Carlsbad, CA, USA). The pool was then diluted to 2 nM, denatured, and then diluted to a final concentration of 4 pM with a 10% PhiX spike for sequencing on the Illumina MiSeq platform.

### Quantitative PCR (qPCR)

A quantitative polymerase chain reaction (qPCR) was performed to measure microbial abundances in units of 16S rRNA gene copies per g wet sediment^54,56^. Each reaction used 5 µl of 2× SYBR Green PCR Master Mix (Applied Biosystems, Carlsbad, CA, USA), 4 µl of template DNA, and 1 µl of primer mix. The 16S rRNA gene 1406F/1525R primer set (0.4 µM, F-GYACWCACCGCCCGT and R-AAGGAGGTGWTCCARCC) was designed to amplify bacterial and archaeal 16S rRNA genes. The rpsL primer pair (0.2 µM, F-GTAAAGTATGCCGTGTTCGT and R-AGCCTGCTTACGGTCTTTA) was used for inhibition control samples to amplify *Escherichia coli* DH10B only. Three dilutions (1/100, 1/500, and 1/1000), as well as an inhibition control (1/100 dilution of *E. coli* DH10B genomic DNA spiked into a 1/100 dilution of the sample), were run in triplicate for each sample and standard. For the standards, *E. coli* DH10B genomic DNA dilutions of 10^−2^, 10^−3^, 10^−4^, 10^−5^, and 10^−6^ of the 20 ng/µl stock solution were used. The qPCRs were run on the ViiA7 Real-Time PCR System (Applied Biosystems, Carlsbad, CA, USA), with cycling conditions as follows: 10 min at 95 °C, 40 cycles of [15 s at 95 °C, then 20 s at 55 °C, then 30 s at 72 °C]. A melt curve was produced by running a cycle of 2 min at 95 °C and a final cycle of 15 s at 60 °C. The cycle threshold (Ct) values were recorded and analyzed using ViiA7 v1.2 software, and 16 S rRNA gene copy numbers were calculated for each sample, accounting for the genome size (4,686,137 bp) and 16S rRNA gene copy number (7) of the standard.

### 16S rRNA gene sequence processing and OTU table generation

Sequences were processed as previously described^54^. Briefly, after demultiplexing by sample, each pair of forward and reverse 16S rRNA gene reads was merged. Sequences were then quality-filtered, and singletons were removed with QIIME^57^ and UPARSE^58^. Dereplicated sequences were then clustered at 97% nucleotide identity using UCLUST v7^59^ to generate a database containing one sequence for each OTU. Sequencing reads from the full dataset were then clustered to the database to generate an OTU table. Each OTU was assigned taxonomy via the Ribosomal Database Project taxonomic classifier^60^, and all OTUs assigned as mitochondria or chloroplasts were removed. The resulting OTU table was rarefied to 3000 16S rRNA gene sequences per sample. Following this OTU table curation, 36 samples across 21 core-depth combinations were retained, of which 30 were replicates (i.e., 15 pairs). For each pair of replicates, each OTU count was averaged (for 14 of 15 pairs, replicates were indistinguishable, Supplementary Fig. 8), and the averages were used for all downstream analyses. For the six samples without successful replicates, OTU counts from a single sample were used.

### Metagenomic sequencing and bioinformatics

Based on preliminary 16S rRNA gene amplicon sequencing data from eight samples (IHM4, IHM36, IHE4, IHE28, MHM4, MHM34, MHE4, and MHE16), three samples with the most distinct microbial communities (IHM4, IHE28, and MHE16) were selected for metagenomic sequencing to maximize recovery of diverse microbial populations. DNA (from the same extractions described above for 16S rRNA gene sequencing) was sent to the Australian Centre for Ecogenomics for metagenomic library construction and sequencing on the Illumina NextSeq platform, as previously described^36,37^. Metagenomic assembly, genome binning to recover microbial MAGs, and annotation (to predict gene functions and reconstruct metabolic pathways) were performed as previously described^61^. Briefly, each metagenome was separately assembled using the CLC de novo assembler v4.4.1 (CLCBio, Denmark), reads were mapped to contigs using BWA v0.7.12-r1039^62^, and the mean coverage of contigs was obtained using the ‘coverage’ command of CheckM v1.0.6^63^. Genomes were binned using MetaBAT v0.26.3^64^ with all five preset parameters (very sensitive, sensitive, specific, very specific, super specific), and genome completeness and contamination were estimated using CheckM^63^. To investigate predicted metabolic functions of interest in the metagenomic data, metagenomic reads with sequence similarity to genes diagnostic of specific metabolic functions (*e.g*., methane monooxygenase, *pmoA*, and methyl-coenzyme M reductase, *mcrA*, indicative of aerobic methane oxidation and methanogenesis, respectively) were identified using hidden Markov models via GraftM v1.0^65^.

### Incubations for CH_4_ production rates

Anaerobic incubations of lake sediment samples were performed to assess rates of production of CH_4_. Four replicate sediment samples (4 ml) from three depths in 2012 (0–5, 10, 20 cm) were collected in the field and immediately sealed in a 120 ml serum vial. The headspace was immediately flushed for 5 minutes with Ultra High Purity (UHP) N_2_ in the field (replacing the headspace 20 times) to establish an anaerobic headspace. The vials were stored in coolers, taken to the research station, flushed again with N_2_ before incubations began in the laboratory, and then stored during the experiment as follows: 2 vials were incubated at 5 °C and 2 vials were held at room temperature (22 °C) for each depth. Consistent with previous incubations from lake sediments^18^, 5 ml of headspace was sampled daily for 5 days and analyzed on a flame ionization GC to determine CH_4_ fluxes. Flux was calculated as a change in headspace concentration over time, then normalized by sediment mass after incubations, when vials were dried and weighed to determine sediment dry weight. We also report data from incubations in 2013 that were treated the same way with samples collected at depths consistent with changes in core sediment transitions: Inre Harrsjön edge: 2.5, 27.5, 47.5 cm; Inre Harrsjön middle: 4.5, 35, 60 cm; Mellersta Harrsjön edge: 7.5, 22.5, 37.5 cm; and Mellersta Harrsjön middle: 2.5, 27.5, 47.5 cm.

### Calculations of depth-resolved fugitive CH_4_

Depth-resolved fugitive CH_4_ (total CH_4_ released from the sediments, including ebullitive and diffusive CH_4_, though ebullition is by far the dominant CH_4_ production pathway in these sediments, accounting for 80–88% of total emissions^12^) was calculated from the concentration and stable carbon isotopic composition of CH_4_ and DIC in sediment porewater^66^. This approach leverages the fact that (1) microbial fermentation and respiration, which generate CO_2_, do not fractionate carbon, while methanogenesis, which generates CH_4_ and CO_2_ (1:1), does fractionate carbon, and (2) DIC largely remains dissolved in water, while dissolved CH_4_ escapes porewater by ebullition. In this framework, the measured isotopic composition of CH_4_ in porewater was used to calculate the fractionation factor associated with methanogenesis, assuming the starting isotopic composition of the substrate matched that measured for organic carbon in the sediment. The model assumes that the products of fermentation, including acetate and/or CO_2_, feed directly into methanogenesis as substrates. While evidence for microbial fractionation of the bulk acetate pool has been demonstrated^67^, we do not have knowledge of the isotopic composition of acetate in our system, and thus we could not use this information to constrain our model. The calculated fractionation factor associated with methanogenesis, along with the measured isotopic composition of DIC in porewater, was used to determine the relative amount of DIC that came from methanogenesis vs. non-fractionating pathways (e.g., fermentation). Because any CO_2_ produced was assumed to stay dissolved in porewater, the relative amount of DIC generated from methanogenesis could be multiplied by the measured concentration of DIC to determine the concentration of CO_2_ and CH_4_ generated through methanogenesis. This generated CH_4_ concentration was larger than the actual measured concentration of CH_4_ in porewater, and the difference between the two was assigned as ‘fugitive’ CH_4_. Calculations assumed that the system was at a steady state.

### Statistics and reproducibility

Homogeneity of regression among groups in Arrhenius plots (Fig. 1 and Supplementary Fig. 2) was tested using pairwise full factorial analyses with JMP statistical software (SAS Institute Inc., Cary, NC). The significance level of the group interaction of lake zone by temperature interval was 0.05 (Supplementary Table 3).

Unless otherwise indicated, other statistical analyses were performed using PRIMER v7^68,69^. The rarefied OTU table was square-root transformed, and Bray-Curtis similarity matrices were generated for sample comparisons and used to make a Principal Coordinates Analysis (PCoA) plot. We used permutational ANOVA (PERMANOVA) to test for significant differences in microbial community composition between categorical groups of samples (e.g., between the two lakes and between the edges and middles of the lakes), and we used Mantel tests with Spearman’s rank correlations to compare microbial community composition (Bray–Curtis similarity matrices) to continuous variables (Euclidean distance matrices), including sediment depth and biogeochemical data. ANOVA and linear regression analyses (Supplementary Tables 9 and 11) were performed with StatPlus v6.1.7.0.

We performed PLSR in the R programming language via the package PLS (function PLSR)^70–72^ to predict measured sediment CH_4_ concentrations from biotic and abiotic variables, similar to our previously described PLSR analyses^36^. Briefly, PLSR models a causal relationship between explanatory variable(s) (in this case, abundances of abiotic measurements and/or microorganisms) and the response variable being predicted (here, measured sediment CH_4_ concentrations). Abiotic variables included all depth-resolved abiotic measurements that were not directly related to CH_4_, as such measurements could be confounding variables in our analysis. The included abiotic variables were: depth, TOC, δ^13^C_TOC_, DIC, S, and TOC:TS. The PLSR analysis yielded Pearson’s product–moment correlations between measured environmental and/or geochemical variables, the abundances of microbial lineages, and the abundances of specific microbial populations. This allowed for a quantification of the added value of microbial abundances in predicting sediment CH_4_ concentrations, relative to predictions from abiotic factors alone. Variance in projection (VIP) scores for each explanatory variable indicate the extent to which that variable was predictive of the response variable (i.e., sediment CH_4_ concentrations), with VIP scores ≥ 1 considered to be highly significant^73^.

### Reporting summary

Further information on research design is available in the Nature Research Reporting Summary linked to this article.

## Supplementary information

Supplementary Information

Dataset 1

Reporting Summary

## Data Availability

Sequencing data are available at NCBI under BioProject PRJNA667178 and also here: https://isogenie-db.asc.ohio-state.edu/datasources#lake_data (from the IsoGenie link, note that the two folders with MAGs are based on initial taxonomy; some MAGs subsequently determined to be archaea are in the bacteria folder and vice versa). NCBI accession numbers are as follows: raw 16S rRNA gene amplicon sequences SRX10114484–SRX10114504, raw metagenomic sequences SRX10063754–SRX10063756, and MAGs JAFNEO000000000–JAFNIC000000000. Other raw data and relevant processed data are provided in supplementary tables and/or associated with prior publications, as cited in the paper. Data underlying Figs. 1–4 can be found as follows: Fig. 1A–C (Supplementary Table 2), Fig. 2C, D (Supplementary Table 5), Fig. 3 (Supplementary Table 10), and Fig. 4A–C (raw data in Supplementary Tables 4–5, relevant processed data in Supplementary Tables 13 and 16).
