# Supplementary material for: Diverse sediment microbiota shape methane emission temperature sensitivity in Arctic lakes
Source: Nat Commun. 2021 Oct 5;12:5815. doi: 10.1038/s41467-021-25983-9 (PMC8492752; doi:10.1038/s41467-021-25983-9)
Supplement: Supplementary file 1 — Supplementary Information [file 41467_2021_25983_MOESM1_ESM.pdf]

Supplementary Information for:

Diverse sediment microbiota shape methane emission temperature sensitivity in Arctic lakes

Joanne B. Emerson[1+*], Ruth K. Varner[2,3*], Martin Wik[4], Donovan H. Parks[5], Rebecca B. Neumann[6], Joel E. Johnson[2], Caitlin M. Singleton[5,++], Ben J. Woodcroft[5], Rodney Tollerson II[1,+++], Akosua Owusu-Dommey[7,++++], Morgan Binder[7,+++++], Nancy L. Freitas[7,++++++], Patrick M. Crill[4], Scott R. Saleska[8], Gene W. Tyson[5], and Virginia I. Rich[1*]

[1]Department of Microbiology, The Ohio State University, 496 W 12th Ave, Columbus, OH 43210, USA.
[2]Department of Earth Sciences, University of New Hampshire, 56 College Road, Durham, NH 03824, USA
[3]Earth Systems Research Center, Institute for the Study of Earth, Oceans and Space, University of New Hampshire, 8 College Road, Durham, NH 03824, USA
[4]Department of Geological Sciences, Stockholm University, Stockholm 106 91, Sweden
[5]Australian Centre for Ecogenomics, School of Chemistry and Molecular Biosciences, University of Queensland, Brisbane 4072, Australia
[6]Civil & Environmental Engineering, University of Washington, 201 More Hall, Box 352700, Seattle, WA 98195-2700
[7]Department of Environmental Science, University of Arizona, AZ 85721, USA
[8]Department of Ecology and Evolutionary Biology, University of Arizona, Tucson, AZ 85721, USA

[+]Current address: Department of Plant Pathology, University of California, Davis, One Shields Ave, Davis, CA 95616 USA
[++]Current address: Center for Microbial Communities, Department of Chemistry and Bioscience, Aalborg University, Aalborg 9220, Denmark
[+++]Current address: Department of Geological and Planetary Sciences, California Institute of Technology, Pasadena, CA, 91106, USA
[++++]Current address: Parkland Hospital, 5200 Harry Hines Blvd., Dallas, TX 75235
[+++++]Current address: John C. Lincoln Health Network, 34975 N North Valley Pkwy Ste 100, Phoenix, AZ 85086
[++++++]Current address: Energy and Resources Group, University of California, Berkeley

*Correspondence to: ruth.varner@unh.edu, rich.270@osu.edu, jbemerson@ucdavis.edu

In this file: Supplementary Discussion, Supplementary Figures 1-8
(Supplementary Tables 1-16 are in a separate Excel file)

Supplementary Discussion

Predicted metabolisms and abundance profiles of MAGs

The Bin 1 metagenome-assembled genome (MAG), from Candidate Phylum Aminicenantes (previously predicted to be fermentative, saccharolytic, and/or aerobic[1]), is predicted to have the capacity for several C1 metabolic processes, including methylotrophy through the assimilation of methylamines, methane-thiols, and/or DMS, and possibly CO_2 fixation through a near-complete Wood-Ljungdahl pathway (WLP; the direction of the WLP is unclear – it may operate in the reverse direction). The dominant Aminicenantes population is also predicted to have a complete pathway for propionate fermentation. The 16S rRNA gene data suggest that the Aminicenantes lineage generally increased in abundance with depth in all four cores, and the population from which the genome was recovered (the most abundant Aminicenantes OTU) was particularly abundant in the deeper regions of the Inre Edge core. Across the three metagenomes, Bin 1 had very high coverage in the Inre Edge 28 cm sample, near 0x coverage in the Inre Middle 4 cm sample, and very low coverage in the Mellersta Edge 16 cm sample.

The Bin 16 MAG was initially taxonomically classified as a member of the archaeal Methanomassiliicoccaceae, presumed in the literature to be a methanogenic lineage, with some populations capable of fermentation in addition to methanogenesis[2]. At 95.2% complete, 6 of the 7 enzymes in the methanogenesis pathway, including all subunits of the methyl coenzyme M reductase (MCR) complex for generating methane, were not detected in this MAG through the analyses associated with the current work, which suggested that this population might lack the capacity for methanogenesis. A follow-up study by Zinke *et al*. considered this MAG (Bin 16 here, SAL16 in Zinke *et al*., NCBI accession #: JAFNFQ000000000) and related lineages in much more detail, and the reader is referred to that paper for more information[3]. In the current study, the 16S rRNA gene data suggested that the Methanomassilicoccaceae (as a lineage) generally maintained similar abundances or increased in abundance with depth in the four cores, and the specific population from which the Bin 16 MAG was recovered (the most abundant Methanomasilliicoccaceae OTU, as classified here, but shown in the subsequent work to be a Thermoplasmata lineage basal to the Methanomassiliicoccaceae) generally increased in abundance with depth and was particularly abundant in the Inre Edge core. Across the three metagenomes, Bin 16 was at moderate (average 18x) coverage depth in the Inre Edge 28 cm sample and at near 0x coverage in both the Inre Middle 4 cm and Mellersta Edge 16 cm samples.

The Bin 19 MAG was classified as a divergent member of the archaeal Thermoplasmatales[4]. Of the predicted proteins encoded by the genome, 38% were hypothetical, meaning that no known homologs with classified functions were recovered in our analyses, suggesting a relatively high amount of previously unknown or divergent functional capacity. The genome indicates the capacity for CO_2 production from formate and amino acid degradation (*e.g.*, complete pathways for L-arginine and L-citrulline degradation to ammonium and CO_2, along with L-histidine degradation). It also encodes gingipain for extracellular peptide degradation, along with genes involved in complex carbon degradation, including beta-xylosidase, alpha-amylases, and chitodextrinase, and it has the capacity for glycogen storage and breakdown. Although the MAG could

not be directly linked to a 16S rRNA gene sequence, the 16S rRNA gene data for the Thermoplasmatales lineage suggested that these organisms generally increased in abundance with depth. Across the three metagenomes, Bin 19 was at approximately 0x coverage in the Inre Edge 28 cm and Inre Deep 4 cm samples and at moderate abundance (average 17x coverage) in the Mellersta Edge 16 cm sample.

Consistent with previous predictions for the Planctomycetes lineage[5], the Phycisphaerae MAG (Bin 34) appears to have the capacity to metabolize a wide variety of complex carbon compounds. Genes encoding the following enzymes were recovered, supporting diverse carbon degradation capacities: trehalose utilization, beta-xylosidases, a variety of glycosyl hydrolases, pectate lyases, beta-xylanase, chitinase, beta-lactamases, cellulose synthase, beta-hexosaminidase, xanthine dehydrogenase, gellan lyase, xylonate dehydratase, beta-galactosidases, alpha-L-fucosidase, alpha-L-arabinofuranosidases, cellobiose-2-epimerases, cellulases, and exo-beta-D-glucosaminidase. With 41% hypothetical proteins, specific metabolic processes are difficult to infer, but the genome also encodes multiple hydrogenases and evidence for C1 metabolism of methylamines, possibly from choline degradation, in addition to some evidence for nitrogen fixation (as previously reported[6]) and sulfur metabolism. Across the three metagenomes, Bin 34 was at moderate abundance (average 12x coverage) in the Inre Edge 28 cm sample and at near 0x coverage in the Inre Middle 4 cm and Mellersta Edge 16 cm samples.

Bin 41, a member of the Syntrophaceae lineage of Deltaproteobacteria, is hypothesized to be a fermenter of diverse carbon compounds, particularly carbon-sulfur compounds, and may have the capacity for respiration (*e.g.*, a predicted fermentation/respiration switch protein was encoded in the genome). Many amino acid transporters and a near-complete pathway for pyruvate fermentation were encoded. Bin 41 was at moderate (average 12x) coverage in the Inre Middle 4 cm sample and near 0x coverage in both the Inre Edge 28 cm and Mellersta Edge 16 cm samples.

Supplementary References

1 Sharon, I. *et al.* Accurate, multi-kb reads resolve complex populations and detect rare microorganisms. *Genome Research*, doi:10.1101/gr.183012.114 (2015).

2 Evans, P. N. *et al.* An evolving view of methane metabolism in the Archaea. *Nature Reviews Microbiology* **17**, 219-232, doi:10.1038/s41579-018-0136-7 (2019).

3 Zinke, L. A. *et al*. Evidence for non-methanogenic metabolisms in globally distributed archaeal clades basal to the Methanomassiliicoccales. *Environmental Microbiology* **23**, 340-357, doi:10.1111/1462-2920.15316 (2021).

4 Lloyd, K. G. *et al.* Predominant archaea in marine sediments degrade detrital proteins. *Nature* 496, 215-218, doi:10.1038/nature12033 (2013).

5 Spring, S., Bunk, B., Spröer, C., Rohde, M. & Klenk, H.-P. Genome biology of a novel lineage of planctomycetes widespread in anoxic aquatic environments. *Environmental Microbiology* **0**, doi:10.1111/1462-2920.14253 (2018).

6 Delmont, T. O. *et al.* Nitrogen-fixing populations of Planctomycetes and Proteobacteria are abundant in surface ocean metagenomes. *Nature Microbiology* **3**, 804-813, doi:10.1038/s41564-018-0176-9 (2018).

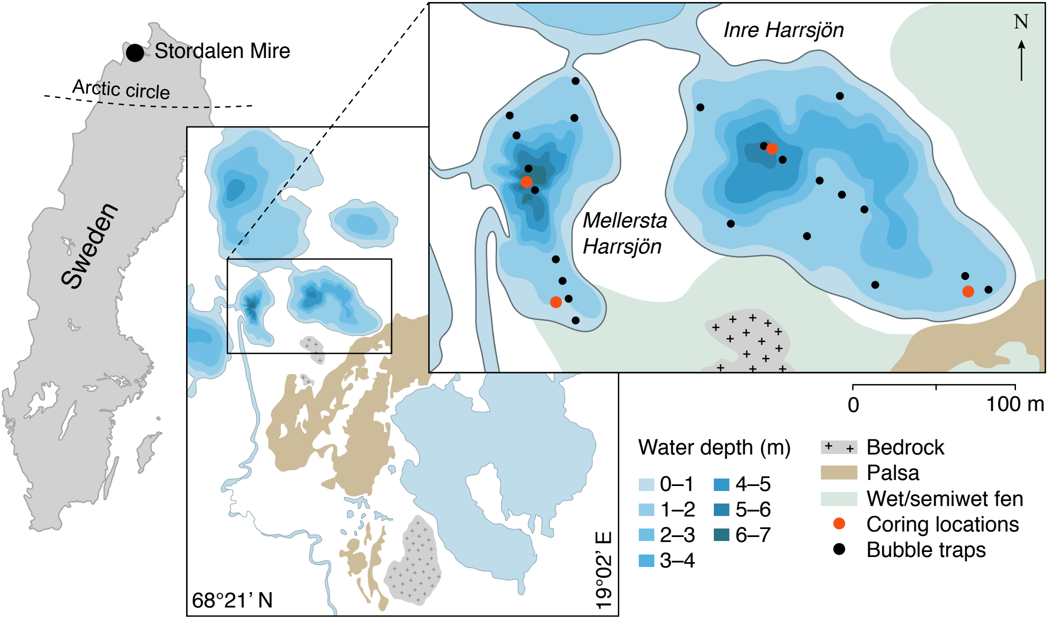

Supplementary Figure 1. Field site, lake, core, and bubble trap locations. Surface bubble traps linked to "middle" cores (deeper waters) are the two adjacent to each middle core (four bubble traps total); the remaining bubble traps were linked to "edge" cores (shallower waters). Adapted from Wik *et al.* 2013, *JGR Biogeosciences*.

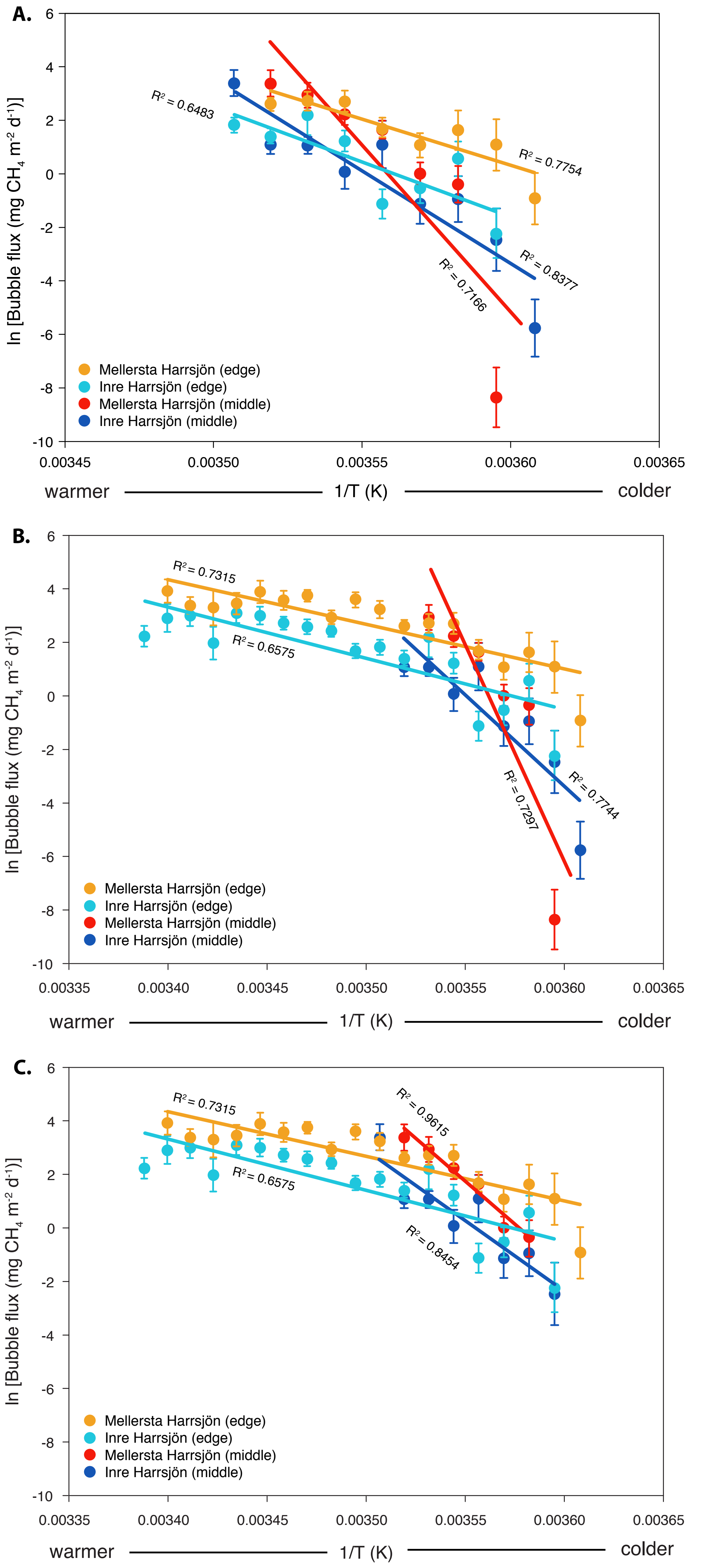

Supplementary Figure 2. Arrhenius plots of temperature and ebullitive methane flux, considering subsets of the data in Figure 1c. Ebullitive CH_4 flux as a function of surface sediment temperature (data were binned in 1 °C intervals; see methods) for the edge versus middle regions of Lake Mellersta Harrsjön and Lake Inre Harrsjön, from June - September 2009 - 2014. Point centers are means, and error bars are 95% confidence intervals. Data are color-coded by lake and by edge (littoral) and middle (pelagic) zones. **A.** Excluding data from lake edges at temperatures not experienced by lake middles (n = 3,258 independent ebullitive CH_4 flux measurements), **B.** Excluding data from lake middles at the highest temperatures that they experienced (n = 5,035 independent ebullitive CH_4 flux measurements), **C.** Excluding data from lake middles at the lowest temperatures that they experienced (n = 5,082 independent ebullitive CH_4 flux measurements).

A.

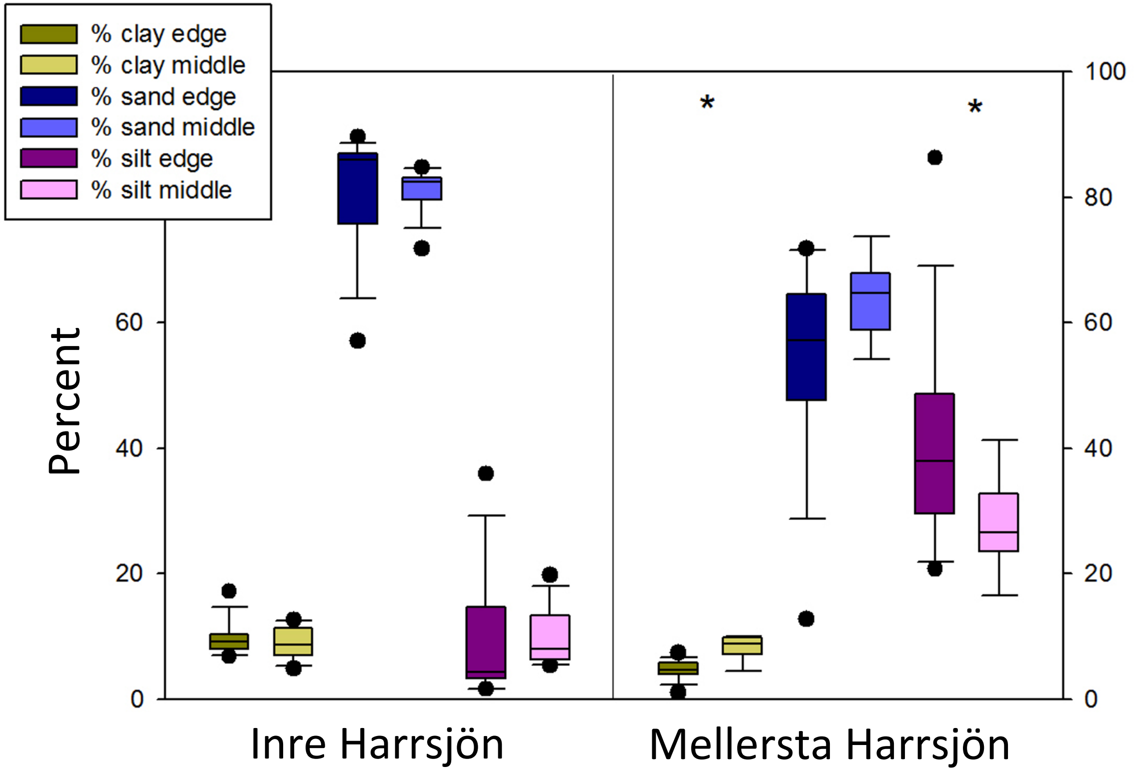

B.

Lake edge vs. middle	clay	sand	silt
Inre Harrsjon	0.324	0.9037	0.8275
Mellersta Harrsjon	**<0.0001**	0.1031	**0.0425**

Supplementary Figure 3. Texture analysis in edges vs. middles of each lake. A. Sand, silt, and clay percentages for each lake, edge vs. middle (n = 51 independent samples: 14 Inre Edge, 13 Inre Middle, 9 Mellersta Edge, 15 Mellersta Middle). Lines in boxes depict the median, boxes indicate the 75th percentile, whiskers 95th percentile, and points are outliers. Asterisks indicate significant edge vs. middle differences (p-values in panel B). **B.** Two-tailed Tukey-Kramer test of texture differences in edges vs. middles. Bold indicates significant differences. P-value for Mellersta clay edge vs. middle is the lowest possible measurement within software constraints (exact p-value not available).

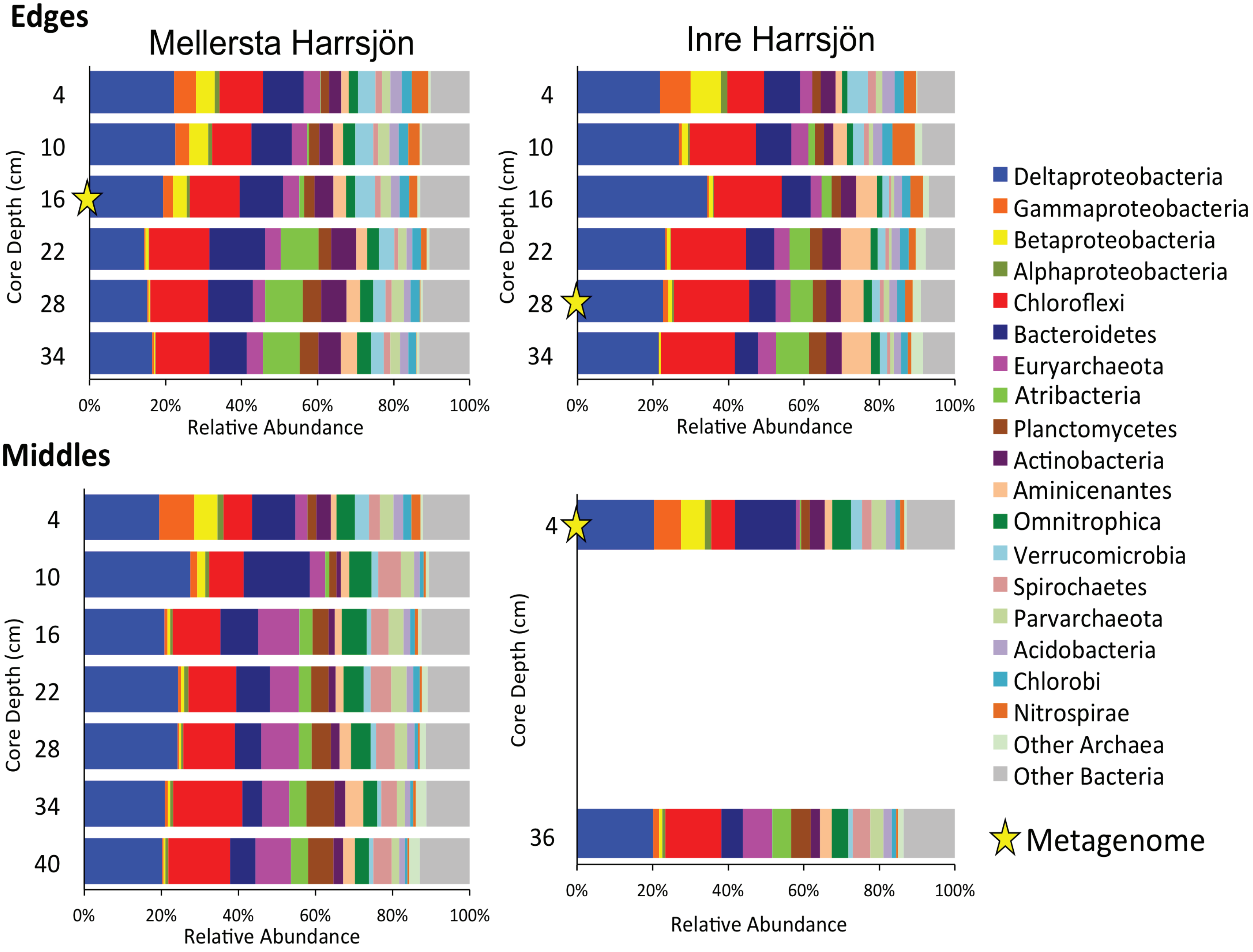

Supplementary Figure 4. Relative abundances of microbial taxa by core and sediment depth. Microbial relative abundances in 16S rRNA gene amplicon sequencing data (Bacteria grouped by phylum, or class for Proteobacteria; Archaea grouped together, see Supplementary Table 5 for OTU abundances).

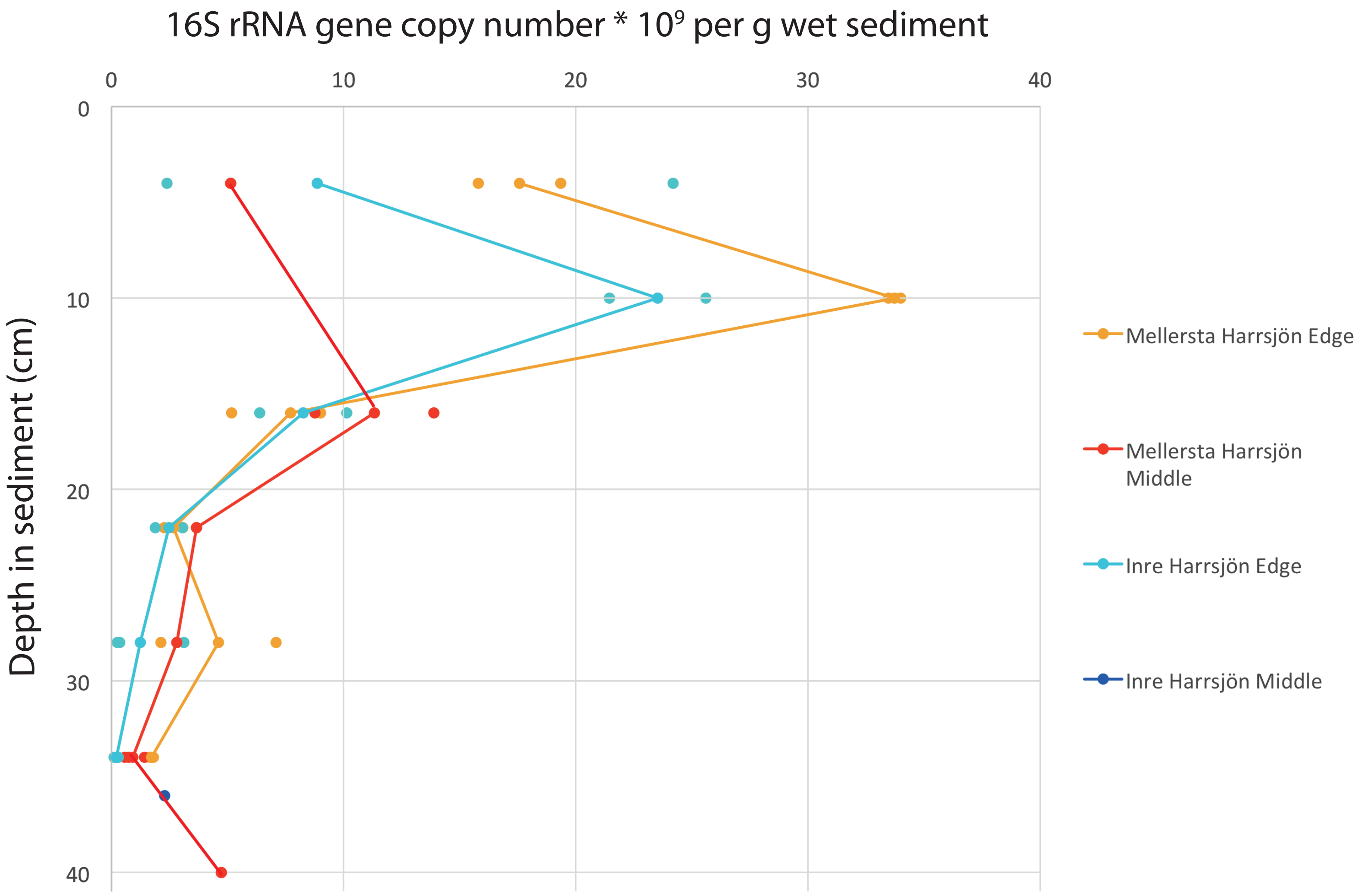

Supplementary Figure 5. Total microbial abundances, as measured by quantitative PCR (qPCR). Points indicate individual measurements, lines indicate trends with depth (line placement is in the middle of replicate measurements where available). 16S rRNA gene copy number is a proxy for abundance (raw data in Supplementary Table 7).

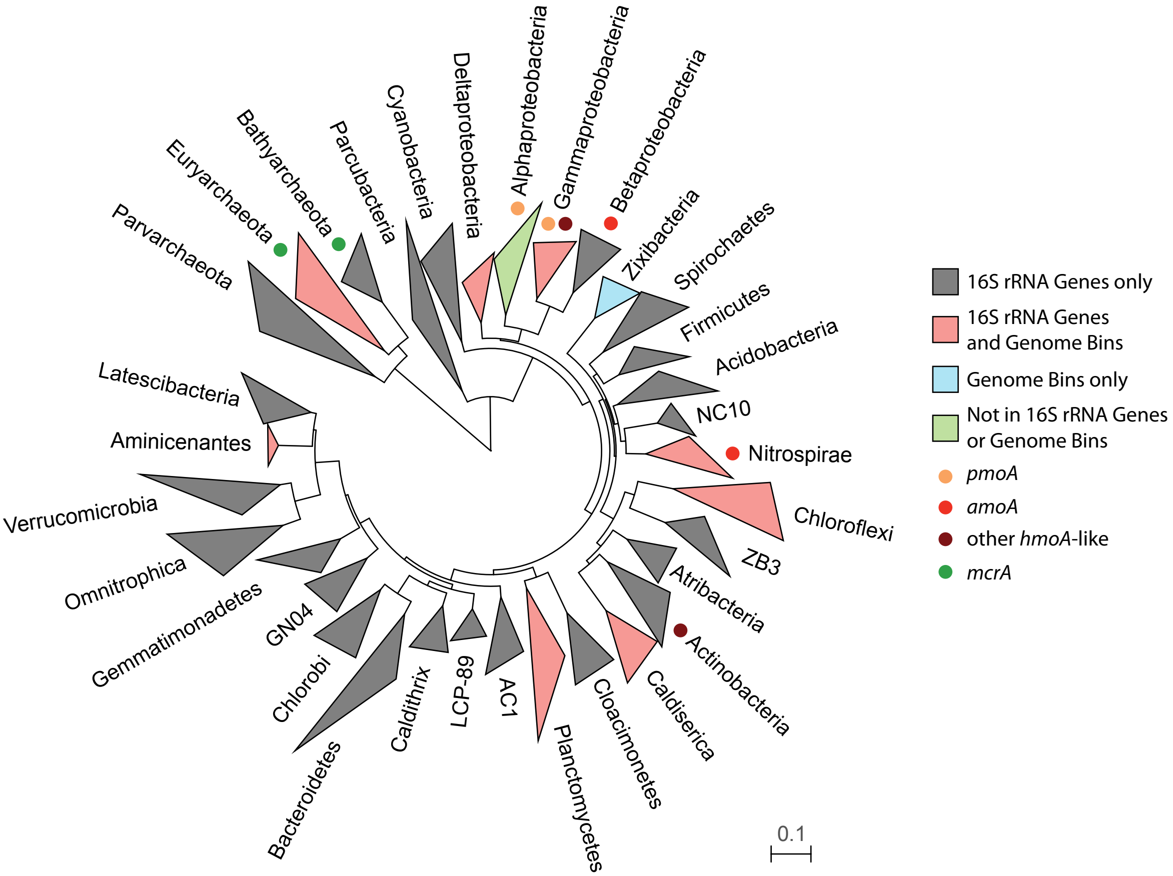

Supplementary Figure 6. Phylogenetic tree of recovered bacterial and archaeal phyla in amplicon and metagenomic data. Tree of representative, publicly available 16S rRNA gene sequences from lineages recovered, colored by their representation in the 16S rRNA gene amplicon data (0.5% relative abundance or higher in any sample) and/or in the 13 draft genomes reconstructed from metagenomic data (>= 50% complete, <= 10% contaminated); colored circles represent *mcrA* (methanogenesis), *pmoA* (methanotrophy), *amoA* (ammonia oxidation), or other *hmoA* -like (*e.g.*, *hmoA*, *pxmA*, function unknown but possibly related to *pmoA* and/or *amoA*) genes recovered from metagenomic reads via the GraftM algorithm; the Alphaproteobacteria did not meet detection thresholds in the 16S rRNA gene amplicon or genomic datasets, but Alphaproteobacteria-like *pmoA* sequences were recovered via GraftM.

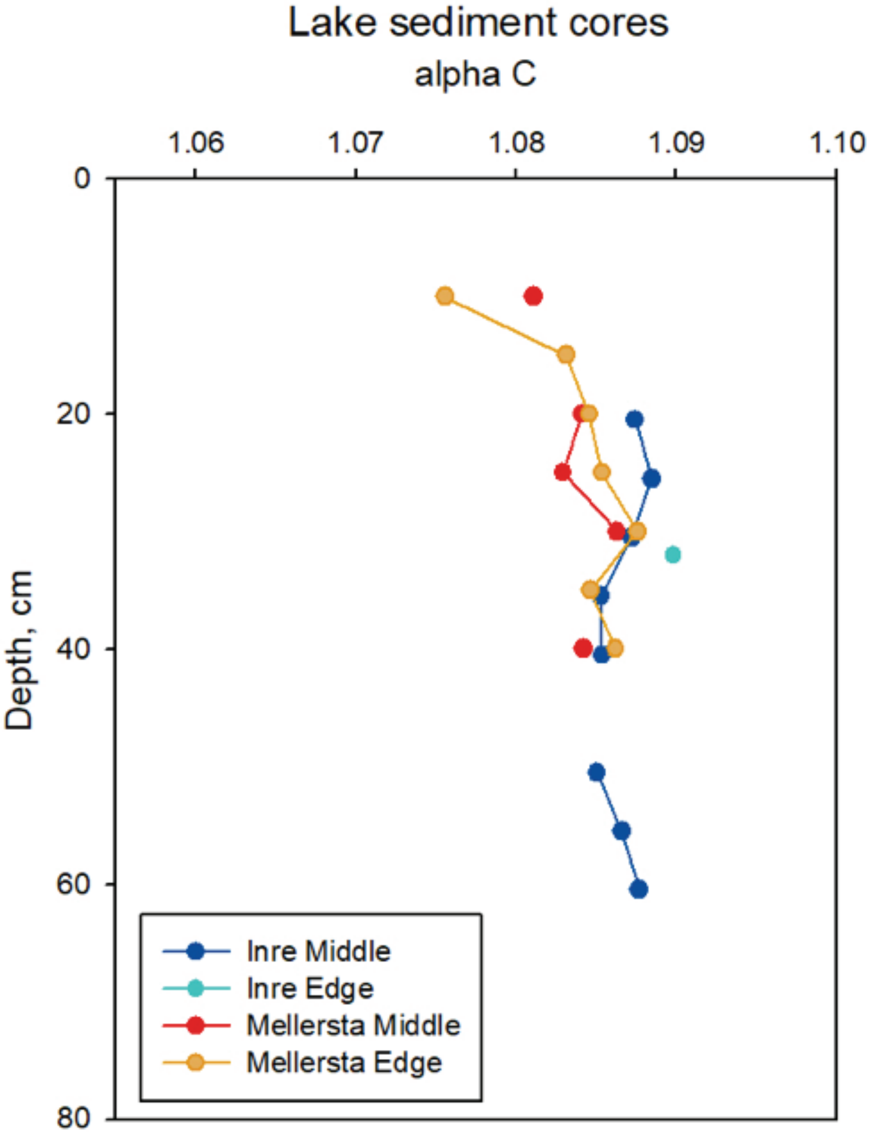

Supplementary Figure 7. Lake sediment porewater alpha values for all four lake locations sampled in 2013. Alpha values are all > 1.07, indicating dominance of hydrogenotrophic methanogenesis in the sediment profiles. For context, alpha values for acetoclastic methanogenesis range from 1.04-1.05, while hydrogenotrophic alpha values range from 1.06-1.09.

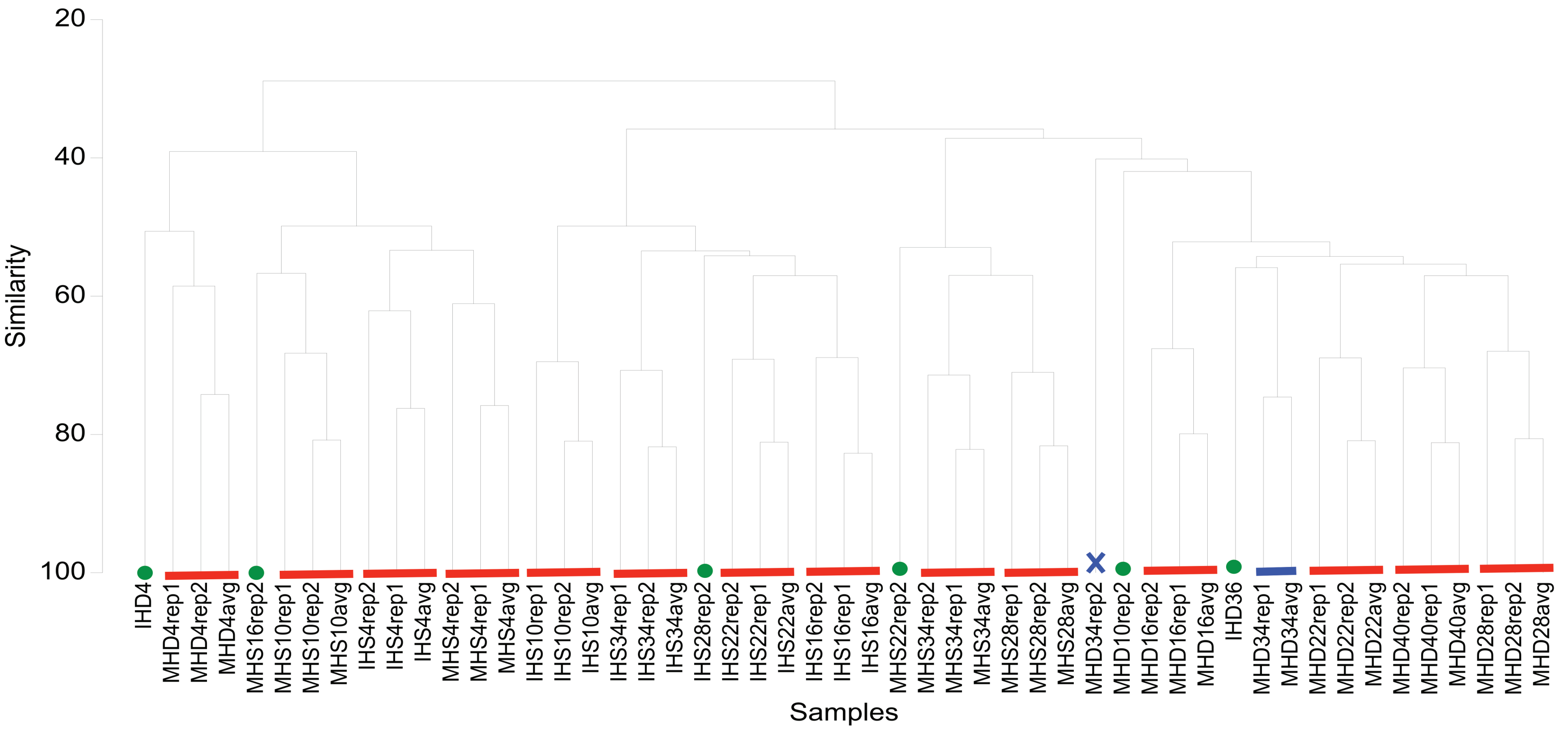

Supplementary Figure 8. Hierarchical clustering analysis of 16S rRNA gene-based microbial community composition in individual samples and in averages of replicates. To assess the extent to which replicates and their averages were consistent enough to use only the average values for each sample in downstream analyses, we prepared this analysis and visual representation of the underlying Bray-Curtis percent similarities (labeled “Similarity”). Red lines indicate replicate and average samples that all cluster closer to each other than to any other sample in the dataset. Green circles indicate samples with no replicates. The blue line and blue X indicate the only replicate samples that were discordant in this comparison.
