# Supplementary material for: Diverse sediment microbiota shape methane emission temperature sensitivity in Arctic lakes
Source: Nat Commun. 2021 Oct 5;12:5815. doi: 10.1038/s41467-021-25983-9 (PMC8492752; doi:10.1038/s41467-021-25983-9)
Supplement: Supplementary file 3 — Reporting Summary [file 41467_2021_25983_MOESM3_ESM.pdf]

nature research

Corresponding author(s): Virginia I. Rich, Ruth K. Varner, Joanne B. Emerson

Last updated by author(s): June 12, 2021

Reporting Summary

Nature Research wishes to improve the reproducibility of the work that we publish. This form provides structure for consistency and transparency in reporting. For further information on Nature Research policies, see Authors & Referees and the Editorial Policy Checklist.

Statistics

For all statistical analyses, confirm that the following items are present in the figure legend, table legend, main text, or Methods section.

n/a	Confirmed	
☐	☒	The exact sample size (*n*) for each experimental group/condition, given as a discrete number and unit of measurement
☐	☒	A statement on whether measurements were taken from distinct samples or whether the same sample was measured repeatedly
☐	☒	The statistical test(s) used AND whether they are one- or two-sided<br>*Only common tests should be described solely by name; describe more complex techniques in the Methods section.*
☐	☒	A description of all covariates tested
☐	☒	A description of any assumptions or corrections, such as tests of normality and adjustment for multiple comparisons
☐	☒	A full description of the statistical parameters including central tendency (e.g. means) or other basic estimates (e.g. regression coefficient) AND variation (e.g. standard deviation) or associated estimates of uncertainty (e.g. confidence intervals)
☐	☒	For null hypothesis testing, the test statistic (e.g. F, t, r) with confidence intervals, effect sizes, degrees of freedom and P value noted<br>*Give P values as exact values whenever suitable.*
☒	☐	For Bayesian analysis, information on the choice of priors and Markov chain Monte Carlo settings
☒	☐	For hierarchical and complex designs, identification of the appropriate level for tests and full reporting of outcomes
☒	☐	Estimates of effect sizes (e.g. Cohen's *d*, Pearson's *r*), indicating how they were calculated

Our web collection on statistics for biologists contains articles on many of the points above.

Software and code

Policy information about availability of computer code

Data collection	This information is provided in the methods section, and briefly, the following data collection software was used: ViiA7 v1.2.
Data analysis	This information is provided in the methods section, and briefly, the following data analysis software/packages were used: ViiA7 v1.2, QIIME, UPARSE, UCLUST v7, CLC de novo assembler v4.4.1, BWA v0.7.12-r1039, CheckM v1.0.6, MetaBAT v0.26.3, GraftM v1.0, JMP, PRIMER v7, StatPlus v6.1.7.0, R packages PLS. All of these tools are publicly available, either online, through prior publications, or as otherwise indicated in the methods section.

For manuscripts utilizing custom algorithms or software that are central to the research but not yet described in published literature, software must be made available to editors/reviewers. We strongly encourage code deposition in a community repository (e.g. GitHub). See the Nature Research guidelines for submitting code & software for further information.

Data

Policy information about availability of data

All manuscripts must include a data availability statement. This statement should provide the following information, where applicable:

- Accession codes, unique identifiers, or web links for publicly available datasets
- A list of figures that have associated raw data
- A description of any restrictions on data availability

Sequencing data are available at NCBI under BioProject PRJNA667178 and also here: https://isogenie-db.asc.ohio-state.edu/datasources#lake_data (note that the two folders with MAGs are based on initial taxonomy; some MAGs subsequently determined to be archaea are in the bacteria folder and vice versa). NCBI accession numbers are as follows: raw 16S rRNA gene amplicon sequences SRX10114484- SRX10114504, raw metagenomic sequences SRX10063754- SRX10063756, and MAGs JAFNEO000000000-JAFNIC000000000. Other raw data and relevant processed data are provided in supplementary tables and/or associated with prior publications, as cited in the manuscript. Data underlying Figures 1-4 can be found as follows: Figure 1A-C (Supplementary Table 2), Figure 2C,D (Supplementary Table 5), Figure 3 (Supplementary Table 10), and Figure 4A-C (raw data in Supplementary Tables 4-5, relevant processed data in Supplementary Tables 13 and 16).

Field-specific reporting

Please select the one below that is the best fit for your research. If you are not sure, read the appropriate sections before making your selection.

☒ Life sciences ☐ Behavioural & social sciences ☐ Ecological, evolutionary & environmental sciences

For a reference copy of the document with all sections, see nature.com/documents/nr-reporting-summary-flat.pdf

Life sciences study design

All studies must disclose on these points even when the disclosure is negative.

Sample size	Sample size was chosen to represent relevant depth ranges from 0-40 cm in lake edges and middles, and the total number of samples for the study was chosen based on the availability of existing cores, depth ranges, and samples at the onset of the study (samples had already been collected previously and were subject to new processing and analysis here). Sample sizes are sufficient for the interpretations made here, as interpretations are restricted to these specific lakes, cores, and sampling dates; generalizations beyond this sample size are speculative and would require further sampling for confirmation.
Data exclusions	No data that met standard threshold criteria were excluded (see methods for thresholds)
Replication	Details on replication are in the methods section and supplementary tables. Briefly, two replicates were included for each 16S rRNA gene sample unless one failed (replicates for six samples failed, see methods and supplementary data), and triplicates were included for qPCR (no replicates failed). Data from triplicate samples were collected for the ex situ incubations (no replicates failed). Raw replicate data are reported in supplementary tables.
Randomization	This is not applicable, as there was no need for randomization; the only experiments reported were sediment incubations, and all treatments were applied to all sediment samples
Blinding	Investigators were not blinded to sample allocation, but, for example, statistical methods were used to determine whether categorical criteria better predicted community composition than random assignment of samples to habitats. It is not practical (due to the relatively small number of samples and labor-intensive nature of the work) for those who collect and process the samples to be different from those who analyze the data, and knowledge of the metadata associated with each sample is required and is standard practice for ecological analyses.

Reporting for specific materials, systems and methods

We require information from authors about some types of materials, experimental systems and methods used in many studies. Here, indicate whether each material, system or method listed is relevant to your study. If you are not sure if a list item applies to your research, read the appropriate section before selecting a response.

Materials & experimental systems

n/a	Involved in the study	
☒	☐	Antibodies
☒	☐	Eukaryotic cell lines
☒	☐	Palaeontology
☒	☐	Animals and other organisms
☒	☐	Human research participants
☒	☐	Clinical data

Methods

n/a	Involved in the study	
☒	☐	ChIP-seq
☒	☐	Flow cytometry
☒	☐	MRI-based neuroimaging
